# Supplementary material for: Allosteric activation of the co-receptor BAK1 by the EFR receptor kinase initiates immune signaling
Source: bioRxiv. 2024 Mar 15:2023.08.23.554490. Originally published 2023 Aug 24. Preprint. [Version 2] doi: 10.1101/2023.08.23.554490 (PMC10473708; doi:10.1101/2023.08.23.554490)

bioRxiv preprint doi: https://doi.org/10.1101/2023.08.23.554490; this version posted March 15, 2024. The copyright holder for this preprint (which was not certified by peer review) is the author/funder, who has granted bioRxiv a license to display the preprint in perpetuity. It is made available under aCC-BY 4.0 International license.

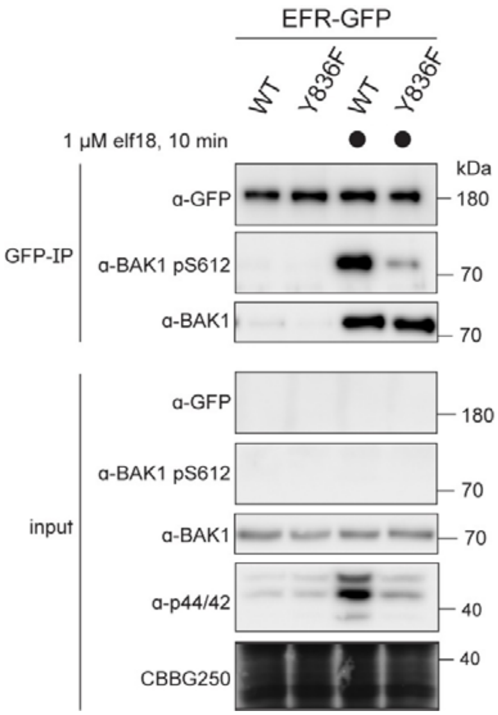

Figure 1 – Supplement 1: EFR^{Y836F} compromises ligand-induced receptor complex activation. Two-week old seedlings were mock treated or treated with 1 μM elf18 for 10 min. Immunoprecipitation was then performed with anti-GFP, and the resulting immunoprecipitates probed for BAK1 S612 phosphorylation. Phosphorylated BAK1 was found to co-immunoprecipitate with WT EFR-GFP but hardly with EFR^{Y836F}-GFP, indicating ligand-induced receptor complex activation.

bioRxiv preprint doi: https://doi.org/10.1101/2023.08.23.554490; this version posted March 15, 2024. The copyright holder for this preprint (which was not certified by peer review) is the author/funder, who has granted bioRxiv a license to display the preprint in perpetuity. It is made available under aCC-BY 4.0 International license.

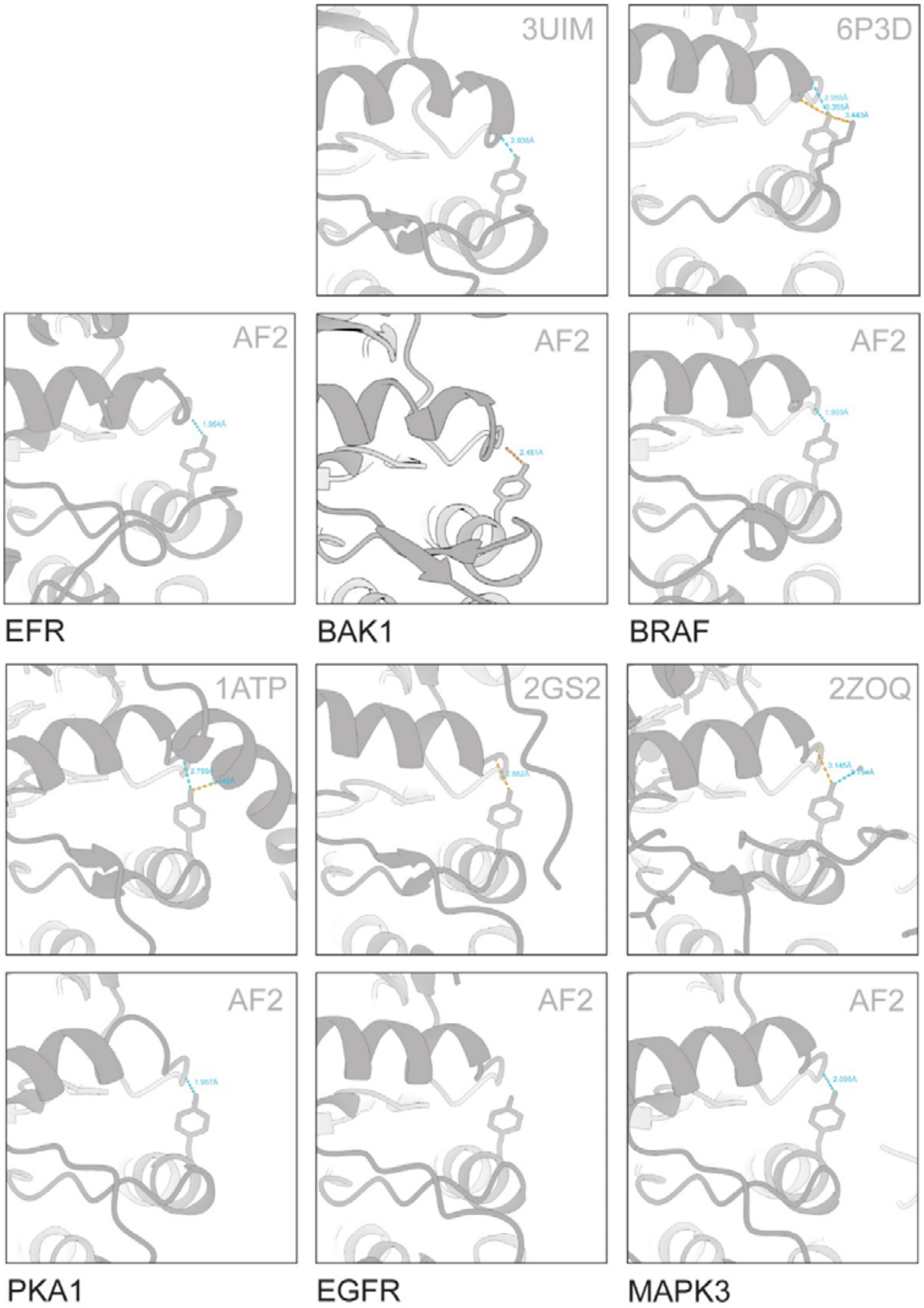

Figure 2 - Supplement 1: VIa-Tyr forms H-bonds with the αC-β4 loop in various predicted and solved structures. Solved structures were retrieved from PDB. AlphaFold2 models for kinases in their active conformation were retrieved from (Faezov and Dunbrack, 2023). BAK1 and EFR models were predicted by AlphaFold2, using the complete intracellular domain. H-bonds were predicted in ChimeraX and distances are indicated.

bioRxiv preprint doi: https://doi.org/10.1101/2023.08.23.554490; this version posted March 15, 2024. The copyright holder for this preprint (which was not certified by peer review) is the author/funder, who has granted bioRxiv a license to display the preprint in perpetuity. It is made available under aCC-BY 4.0 International license.

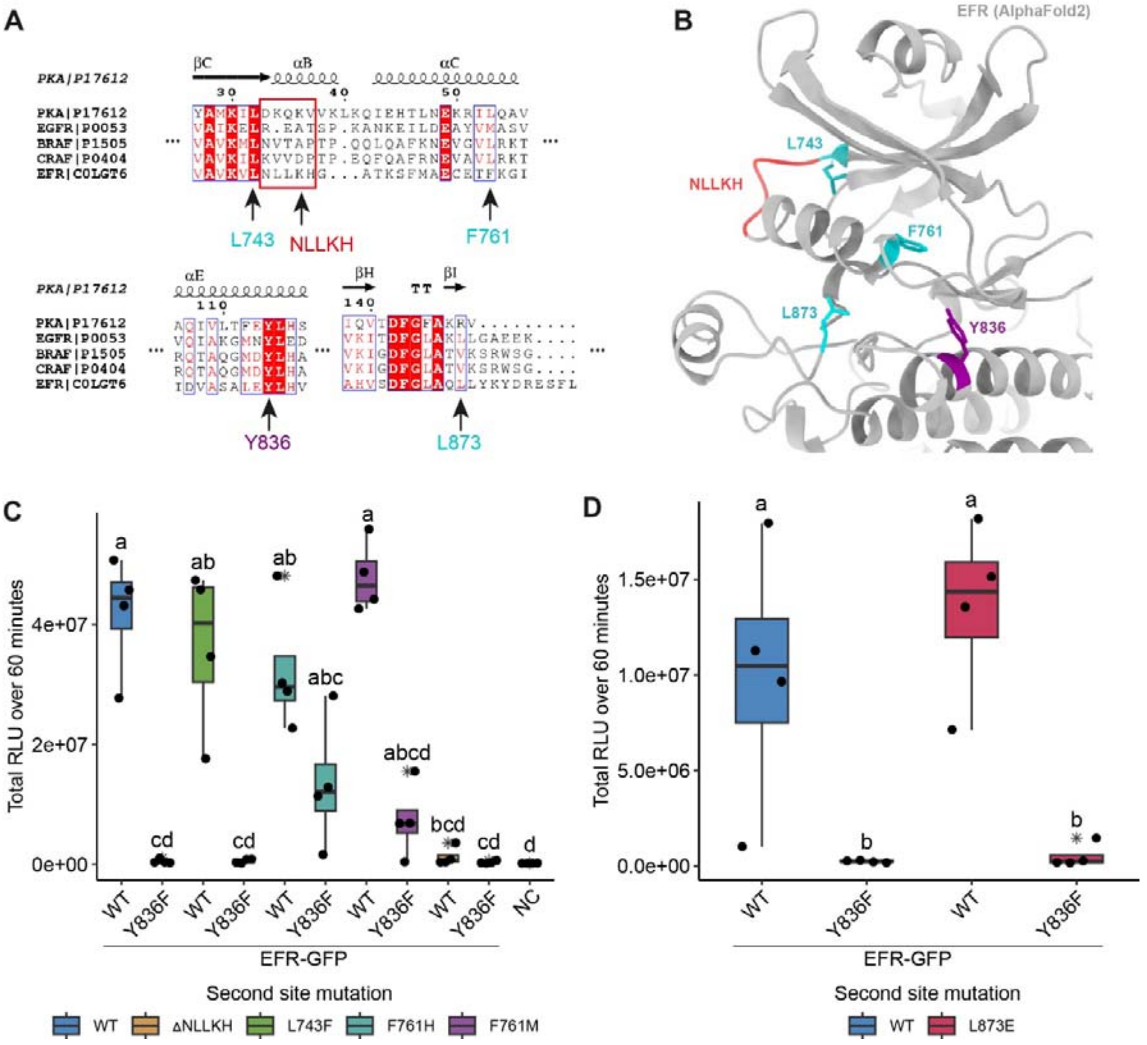

Figure 2 – Supplement 2: Rational design of activating mutations in EFR and screen for functional recovery of EFR^{Y836F}. A) Alignments of EFR with human kinases containing oncogenic, kinase activating mutations that stabilize the αC-helix-in active-like conformation (described in Foster et al., 2016; Hu et al., 2015). Homologous sites in EFR are indicated by arrows with the residue number. B) Structural model of the EFR kinase domain from AlphaFold2 with homologous sites identified in the sequence alignment from A highlighted in teal (missense mutation) or red (deletion). EFR Y836 at the C-terminal end of the αE-helix is colored purple. C) Screening of the homology-based putatively activating EFR mutations for restoration of EFR^{Y836F} function in *N. benthamiana.* All putative activating mutations were functional at WT-like level except EFR$^{\Delta NLLKH}$. Only EFR$^{F761[HM]}$ could functionally recover EFR^{Y836F} as the oxidative burst was partially restored. D) EFR^{L873E} showed a WT-like oxidative burst but EFR$^{L873E/Y836F}$ did not restore the oxidative burst. Outliers are in indicated

bioRxiv preprint doi: https://doi.org/10.1101/2023.08.23.554490; this version posted March 15, 2024. The copyright holder for this preprint (which was not certified by peer review) is the author/funder, who has granted bioRxiv a license to display the preprint in perpetuity. It is made available under aCC-BY 4.0 International license.

by asterisk in addition to the outlier itself and are included in statistical analysis; Statistical test: Kruskal-Wallis test ($p = 9.319*10^{-6}$ in C, $p = 0.01242$ in D), Dunn's post-hoc test with Benjamin-Hochberg correction ($p <= 0.05$) Groups with like lowercase letter designations are not statistically different.

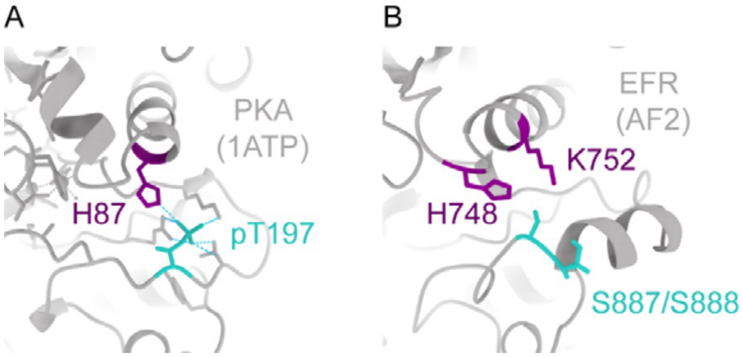

Figure 2 Supplement 3: EFR A-loop phosphorylation sites may coordinate with basic residues from the β3-αC loop and αC-helix. A) In PKA (1ATP), the A-loop phosphorylation on T197 coordinates with H87 from the αC-helix. B) In EFR (AlphaFold2 (AF2) model), there are two basic residues extending downwards from β3-αC loop (H748) and αC-helix (K752) that may coordinate with A-loop phosphorylation on S887 or S888.

bioRxiv preprint doi: https://doi.org/10.1101/2023.08.23.554490; this version posted March 15, 2024. The copyright holder for this preprint (which was not certified by peer review) is the author/funder, who has granted bioRxiv a license to display the preprint in perpetuity. It is made available under aCC-BY 4.0 International license.

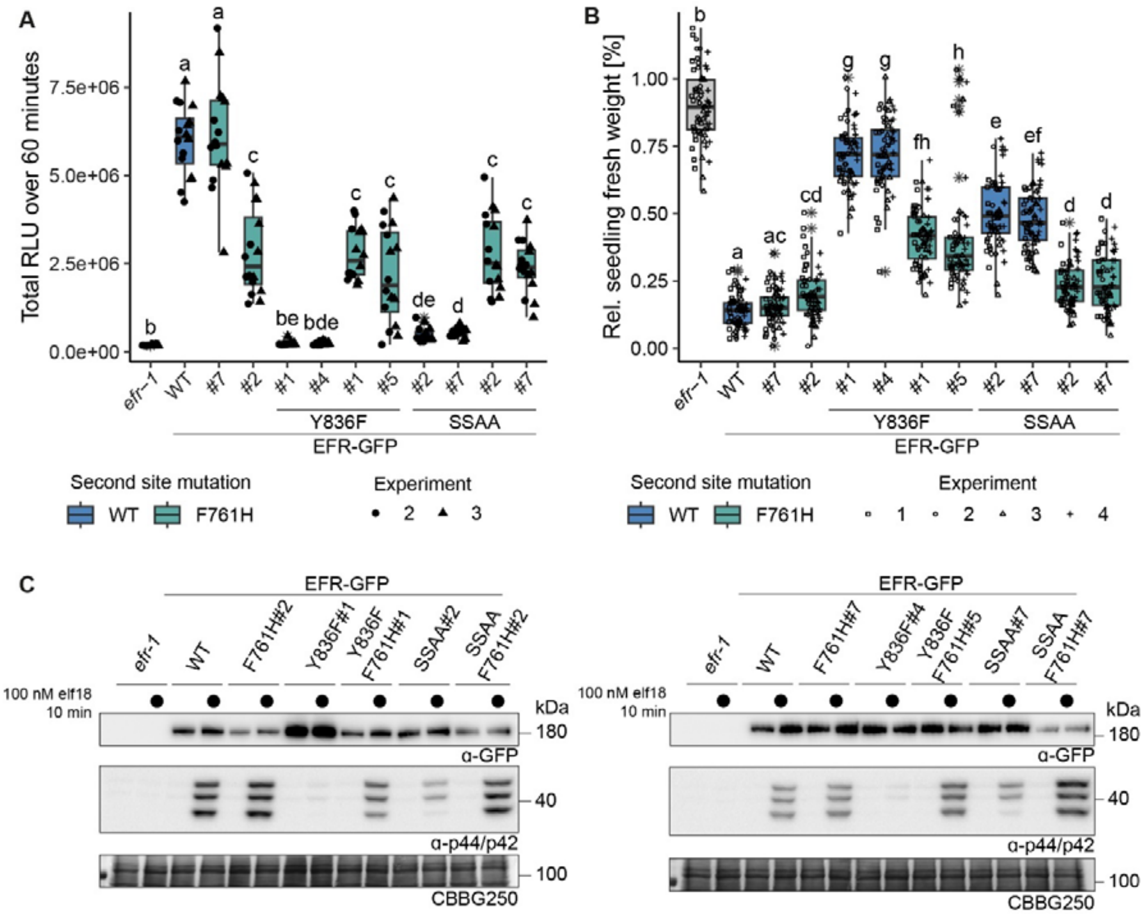

Figure 3 – Supplement 1: Multiple immune signaling branches are partially restored in EFR$^{F761H/Y836F}$ and EFR$^{F761H/SSAA}$. Stable transgenic complementation lines in the Arabidopsis *efr-1* background were generated and physiological experiments conducted in the T3 generation (except for EFR$^{F761H\ /Y836F}$#5, which is a double insertion line in T2 generation). A) In the oxidative burst assay, EFR F761H restored oxidative burst in EFR$^{F761H/Y836F}$ and EFR$^{F761H/SSAA}$ complementation lines. Two independent experiments were merged into one graph as WT controls showed comparable total oxidative burst. A third independent experiment was performed with similar results. Outliers are indicated by an additional asterisk and included in statistical analysis. Statistical test: Kruskal-Wallis test ($p < 2.2*10^{-16}$), Dunn's post-hoc test with Benjamin-Hochberg correction ($p <= 0.05$) Groups with like letter designations are not statistically different. Alike oxidative burst assays, EFR F761H restored SGI (B) and MAPK activation (C) in EFR$^{F761H/Y836F}$ and EFR$^{F761H/SSAA}$ complementation lines. For SGI assays, four independent experiments wtih 5 nM elf18 treatment are shown. Outliers are indicated by an additional asterisk and included in statistical analysis. Statistical test: Kruskal-Wallis test ($p < 2.2 *10^{-16}$), Dunn's post-hoc test with Benjamin-Hochberg correction ($p <= 0.05$) Groups with like letter designations are not statistically different. For MAPK activation assays, a representative experiment is shown. Similar results were obtained in three more experiments.

bioRxiv preprint doi: https://doi.org/10.1101/2023.08.23.554490; this version posted March 15, 2024. The copyright holder for this preprint (which was not certified by peer review) is the author/funder, who has granted bioRxiv a license to display the preprint in perpetuity. It is made available under aCC-BY 4.0 International license.

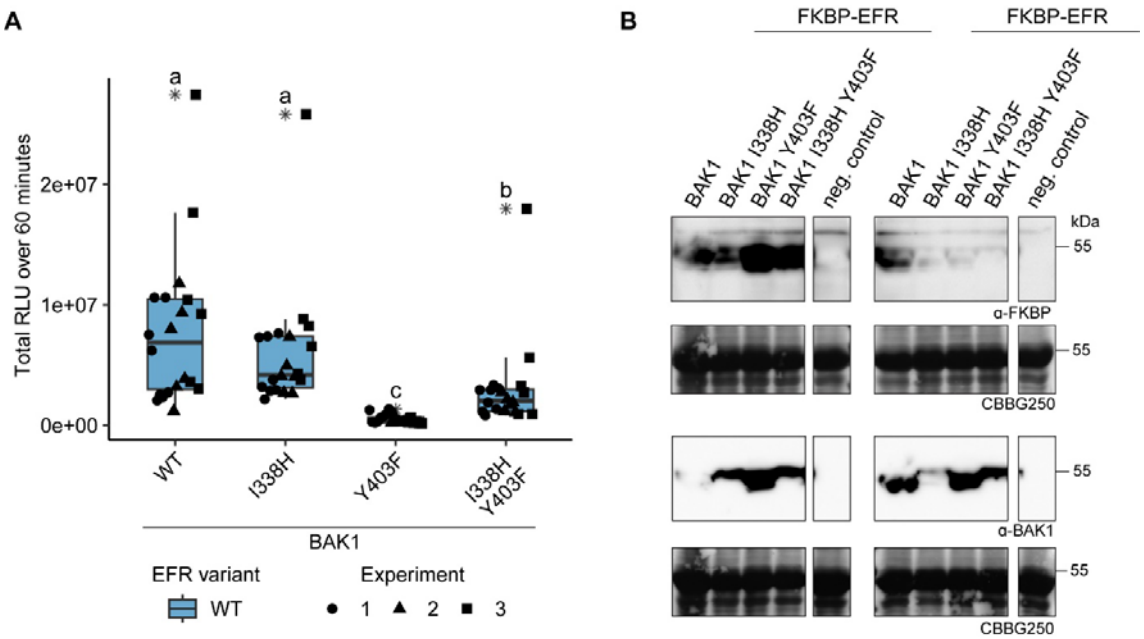

Figure 4 – Supplement 1: Function of BAK1 Y403F is partially recovered by a regulatory spine mutation (I338H) that stabilizes the active-like conformation. A) The RiD system was utilized to test the recovery of BAK1 Y403F by transient expression in *N. benthamiana*. The Y403F mutation in BAK1 diminished the oxidative burst, whereas BAK1 I338H displayed near WT-like responses. Combining the I338H and Y403F mutations, however, led to a partial recovery of oxidative burst. Outliers are indicated by an additional asterisk and included in statistical analysis. Statistical test: Kruskal-Wallis test ($p < 2.247 * 10^{-11}$), Dunn's post-hoc test with Benjamin-Hochberg correction ($p <= 0.05$) Groups with like letter designations are not statistically different. B) SDS-PAGE analysis of protein levels in experiments 2 and 3 of A is shown. Protein accumulation data for experiment 1 was not collected.

bioRxiv preprint doi: https://doi.org/10.1101/2023.08.23.554490; this version posted March 15, 2024. The copyright holder for this preprint (which was not certified by peer review) is the author/funder, who has granted bioRxiv a license to display the preprint in perpetuity. It is made available under aCC-BY 4.0 International license.

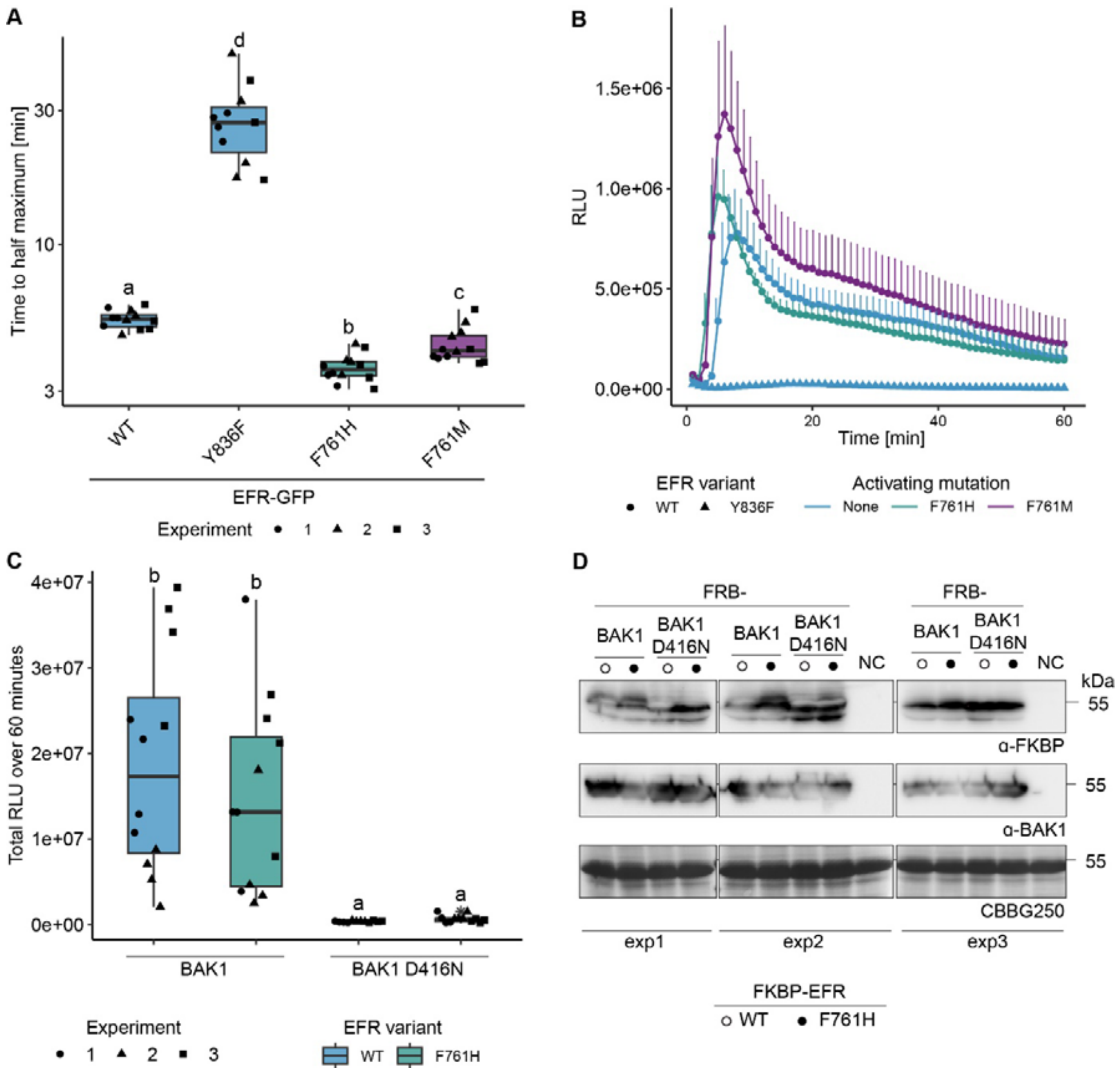

Figure 4 - Supplement 2: EFR F761H accelerates the onset of the oxidative burst but requires the catalytic activity of BAK1. A) Quantification of the time until the oxidative burst reaches its half maximum from experiments presented in Figure 2B. Both putative activating mutations, F761H and F761M accelerate the onset of the oxidative burst. B) Time resolved oxidative burst assay. Presented curves are from replicate number three as a representative example. Graphs in A and B are based on data presented in Figure 2B. Error bars represent standard error of the mean. C) EFR F761H requires the catalytic activity of BAK1 to induce the oxidative burst. Data from three independent experiments is merged in one graph. D) Protein accumulation of the RiD-tagged protein related to panel C. Statistical analysis in A and C: Outliers are indicated by an additional asterisk and included in statistical analysis. Statistical test: Kruskal-Wallis test ($p = 1.686*10^{-8}$ in A, $p = 5.89910^{-8}$ in C), Dunn's post-hoc test with Benjamin-Hochberg correction ($p <= 0.05$) Groups with like letter designations are not statistically different.

bioRxiv preprint doi: https://doi.org/10.1101/2023.08.23.554490; this version posted March 15, 2024. The copyright holder for this preprint (which was not certified by peer review) is the author/funder, who has granted bioRxiv a license to display the preprint in perpetuity. It is made available under aCC-BY 4.0 International license.

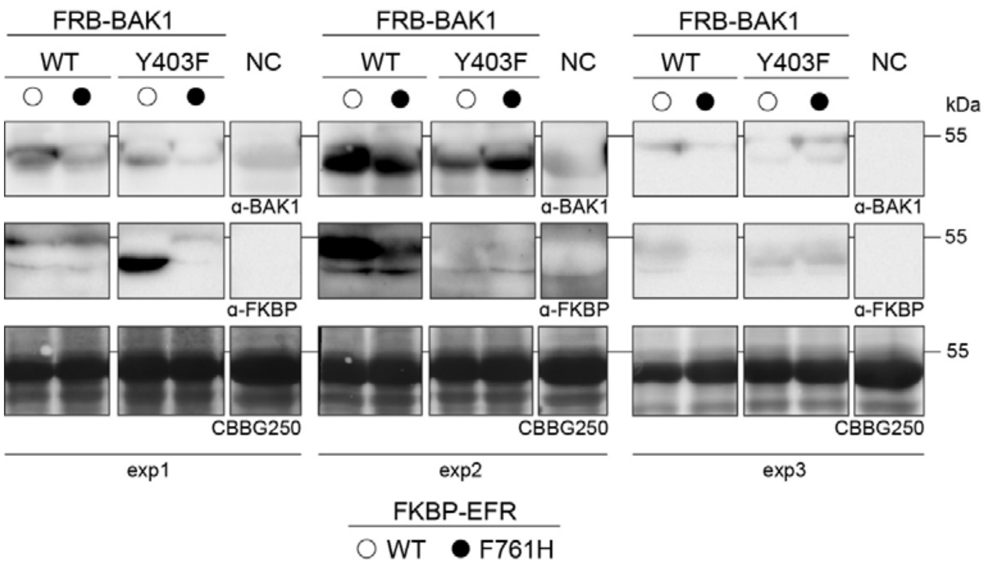

Figure 4 – Supplement 3: Protein accumulation for the oxidative burst assay in Figure 4. Leaf discs were collected after the oxidative burst assay and protein were extracted by boiling in SDS-loading buffer followed by immunoblotting. Non-infiltrated leaf discs served as negative control.

bioRxiv preprint doi: https://doi.org/10.1101/2023.08.23.554490; this version posted March 15, 2024. The copyright holder for this preprint (which was not certified by peer review) is the author/funder, who has granted bioRxiv a license to display the preprint in perpetuity. It is made available under aCC-BY 4.0 International license.

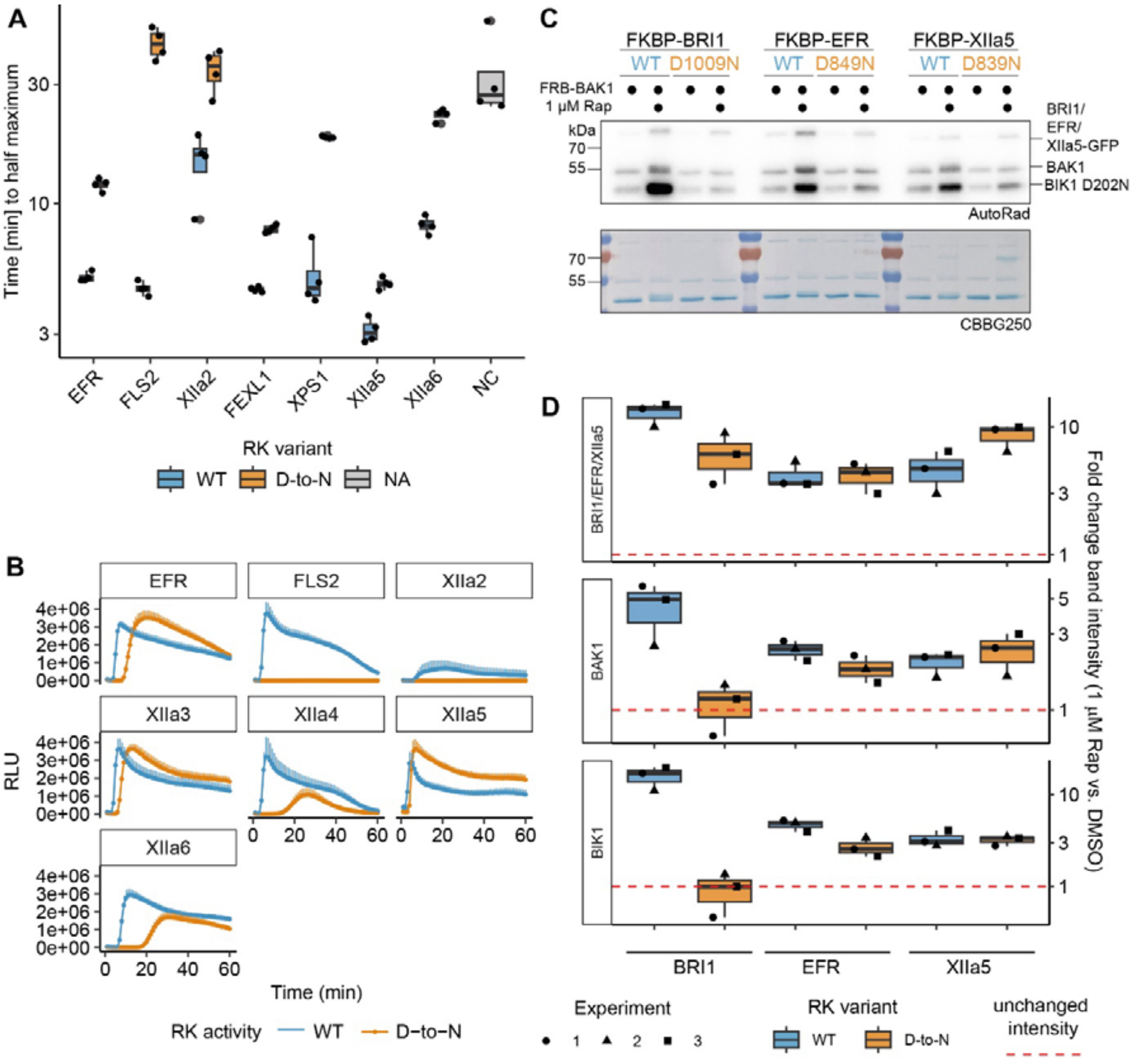

Figure 5 – Supplement 1:XIIa5^{D839N} exhibits largely XIIa5WT-like characteristics. A,B) Catalytic site mutation of XIIa5 exhibited the least delayed onset of oxidative burst. In A, quantification of the time to reach the half maximum of the oxidative burst is shown. The underlying data are the same as used for total oxidative burst in the main figure. In B, the actual oxidative burst curves are presented as average of six individual plants transiently expressing the indicated chimeric protein. Error bars represent standard error of the mean. C) A catalytic site mutation of XIIa5 did not negatively affect BIK1 trans-phosphorylation or BAK1 autophosphorylation. Experiments were performed as described in Figure 1. D) Quantification of band intensities on autoradiographs from three independent experiments are shown. BRI1^{D1009N} and EFR^{D849N} displayed results similar to Figure 1B,C. In contrast to

bioRxiv preprint doi: https://doi.org/10.1101/2023.08.23.554490; this version posted March 15, 2024. The copyright holder for this preprint (which was not certified by peer review) is the author/funder, who has granted bioRxiv a license to display the preprint in perpetuity. It is made available under aCC-BY 4.0 International license.

EFR^{D849N}, for which BIK1 and BAK1 relative band intensities slightly decreased compared to wild type EFR, $BIK1^{D202N}$ and BAK1 relative band intensities were wild-type-like for $XIIa5^{D839N}$.

Figure 5 – Supplement 2: Protein accumulation of EFR-XIIa chimeras in *N. benthamiana*. All constructs exhibited detectable protein accumulation in transiently transformed *N. benthamiana* leaves. Similar protein accumulation was observed in 3 replicates for A. Protein accumulation for constructs in B was tested once.

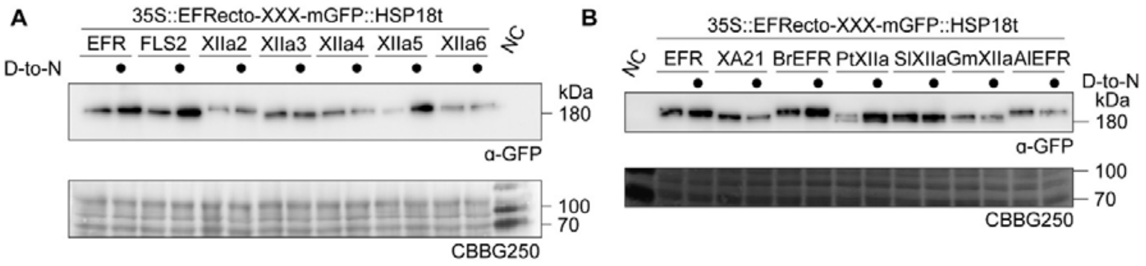

Supplement: 1 [file NIHPP2023.08.23.554490V2-supplement-1.pdf]
